# Dietary factors and the risk of lung cancer by epidermal growth factor receptor mutation status and histological subtypes

**DOI:** 10.3389/fpubh.2022.1079543

**Published:** 2022-12-02

**Authors:** Xin Yin, Gillianne Geet Yi Lai, Adeline Seow, Daniel Shao Weng Tan, Darren Wan-Teck Lim, Wei Jie Seow

**Affiliations:** ^1^Saw Swee Hock School of Public Health, National University of Singapore and National University Health System, Singapore, Singapore; ^2^Division of Medical Oncology, National Cancer Centre Singapore, Singapore, Singapore; ^3^Duke-NUS Medical School, National University of Singapore, Singapore, Singapore; ^4^Department of Medicine, Yong Loo Lin School of Medicine, National University of Singapore and National University Health System, Singapore, Singapore

**Keywords:** diet, lung cancer, *EGFR*, case-control, non-small cell lung cancer

## Abstract

**Background:**

Previous studies have reported differential associations of certain dietary factors such as soy consumption by epidermal growth factor receptor mutant (*EGFR* +) subtype of non-small cell lung cancer (NSCLC). However, whether the other dietary factors including meat, fruits, and vegetables have differential risks on different histological and molecular subtypes of lung cancer remains unclear. Therefore, we conducted a case-control study to evaluate these associations.

**Methods:**

A total of 3,170 cases and 4,238 controls from three different studies (Genes and Environment in Lung Cancer Study, Lung Cancer Consortium Singapore Study, and Multi-ethnic Cohort Study) were included. Information on demographics, lifestyle, and dietary consumption was obtained using questionnaires. Diet was assessed by using the number of standard servings of each item consumed per week. Multivariable logistic regression was used to estimate odds ratios (ORs) and 95% confidence intervals (CIs) for the association between meat, vegetables, and fruits consumption with lung cancer risk after adjusting for potential confounders.

**Results:**

We identified a significant inverse association between higher consumption of fruits and the risk of lung cancer (2nd tertile: OR = 0.54, 95%CI = 0.46–0.65; 3rd tertile: OR = 0.77, 95%CI = 0.65–0.91), compared with the lower (1st tertile) consumption of fruits. Higher vegetable consumption was significantly associated with a lower risk of *EGFR* + lung cancer (OR = 0.69, 95% CI = 0.54–0.88), however, this association was not significant among *EGFR* wild-type (−) lung cancer. Conversely, higher consumption of total meat (OR = 2.10, 95%CI = 1.58–2.79) was significantly associated with higher lung cancer risk, as compared with the lower consumption group.

**Conclusions:**

Differential associations between vegetable consumption with *EGFR* mutation status in NSCLC were found. Further prospective studies are warranted to assess this association and elucidate the biological mechanisms.

## Introduction

Lung cancer is the leading cause of cancer death and disability-adjusted life-years (DALYs) worldwide (1–3). In 2019, there were an estimated 2.26 million incident cases of lung cancer and 2.04 million deaths that occurred globally, accounting for 45.9 million DALYs (3). Non–small cell lung cancer (NSCLC), including adenocarcinoma, squamous cell carcinoma, and large cell carcinoma histologic subtypes, accounted for approximately more than 80% of lung cancers (4). Different histological types of lung cancer have different age and sex distribution, smoking status, clinical performance status, biological pathways, and overall survival rate (5, 6). Epidermal growth factor receptor (*EGFR*), a transmembrane protein with tyrosine kinase activity, is one of the most well-documented and investigated pathways in NSCLC (7), and has been identified as an oncogenic driver, playing an important role in regulating the proliferation, survival, and differentiation of tumor cells (7, 8). *EGFR* mutations are found in approximately 60% of never-smoking Asian patients with adenocarcinomas compared to 5–10% in Caucasians, and thus represent a significant proportion of NSCLC in our local context (9, 10).

The associations between dietary factors and lung cancer risk have been explored by previous studies. A healthy dietary pattern was associated with a lower risk of lung cancer (11). For example, fruits and vegetables are a rich source of vitamin C, vitamin E, carotenoids, and other micronutrients, which are previously reported to have a protective association with the risk of lung cancer and other cancers (12). A meta-analysis showed that the highest consumption group of fruits and vegetables was inversely associated with the risk of lung cancer, as compared with the lowest consumption group (13). In contrast, the literature on the association between meats and lung cancer was conflicting. Some studies suggested that red meat and processed meat were both positively associated with the risk of lung cancer (14, 15), especially among never-smokers (16, 17). However, other studies revealed either a null association or a statistically significant inverse association between meat and the risk of lung cancer (18, 19). When stratified by the types of meat, a meta-analysis demonstrated an inverse association between poultry consumption and lung cancer, based on 11 studies, but not for total white meat or fish (16). A similar trend was identified among never-smokers; higher consumption of red meat was found to be associated with an increased risk of lung cancer, and no significant associations were observed between other types of meat and lung cancer risk (20).

Associations between dietary factors and lung cancer risk have been shown to vary by histological and molecular subtypes. Some studies demonstrated that when stratified by histological subgroups of lung cancer, including adenocarcinoma, squamous cell carcinoma, and large cell carcinoma, the above-mentioned positive or inverse associations became statistically insignificant (13, 14). A previous study in Japan has reported differential associations of soy consumption by *EGFR* lung cancer subtypes (21); the protective effect of soybean products was found only among *EGFR* mutated lung cancer. Another study demonstrated that an alkaline diet prolonged overall survival among NSCLC patients with *EGFR* mutations (22). Furthermore, anthocyanidin extracted from fruits and vegetables was identified as an effective inhibitor of *EGFR* mutated cancers (23), and a low-protein diet combined with an *EGFR* inhibitor was reported to be a promising cancer therapy method (24). These studies demonstrated the potential differential associations between some dietary factors and *EGFR* lung cancer subtypes. *EGFR* can be abnormally activated by various mechanisms, and constitutive *EGFR* tyrosine kinase activation caused by mutations in the tyrosine kinase binding pocket is one of the key targets of specific small molecule inhibitors (25). *EGFR* tyrosine kinase inhibitors have been found to significantly improve outcomes in patients with advanced NSCLC that contain an activating *EGFR* mutation compared with platinum-based chemotherapy (26–29). However, whether other dietary factors are differentially associated with different histological and molecular subtypes of lung cancer remains unclear (13), particularly among the Asian population (21).

In this study, we evaluated the association between the consumption of meats, vegetables, and fruits with the risk of lung cancer by histological and molecular subtypes among Asians.

## Materials and methods

### Study population

A total of three studies were included: the Genes and Environment in Lung Cancer (GEL) Study (case-control), Lung Cancer Consortium Singapore (LCCS) Study (case-only), and the Multi-ethnic Cohort Study (MEC) study (cohort). The LCCS is a case-only study of lung cancer with clinical data from three hospitals, including Singapore General Hospital (SGH), Changi General Hospital (CGH), and the National Cancer Center Singapore (NCCS). A total of 3,245 lung cancer patients, including 1,252 females and 1,993 males, with a diagnosis mean age of 63.4 years were included in the LCCS study between 2007 and 2017 (30).

The GEL study is a hospital-based case-control study of 815 controls and 399 cases recruited from 2005 to 2008, from Singapore public hospitals, including SGH, CGH, National University Hospital (NUH), and Tan Tock Seng Hospital (TTSH) (31, 32). Controls and cases were recruited from the same hospitals and frequency-matched by 10-year age groups. Controls were selected within one month after the date of diagnosis of the corresponding cases.

The MEC is a cohort study that was formed by combining two existing population-based studies, the Singapore Prospective Study Program (SP2) and the Singapore Cardiovascular Cohort Study (SCCS2), with additional recruitment of participants from 2007 to 2010 (33). The baseline of the MEC study recruited 13,777 participants. After excluding those who have been diagnosed with cancer at the baseline, a total of 13,149 cancer-free controls were included.

Lung cancer subtypes were extracted from medical records. Lung tumor tissues from the LCCS study were tested for their *EGFR* mutation status (mutation/+, or wildtype/-) using direct Sanger sequencing, or the real-time polymerase chain reaction (PCR) test. All *EGFR* tests were done at the Singapore General Hospital. Lung cancer cases in this current study were obtained from the LCCS and GEL studies. Healthy controls were obtained from the GEL and MEC baseline studies. Therefore, a total of 3,644 lung cancer cases and 13,964 controls were included in our study.

This current study of using three datasets was approved by the National University of Singapore Institutional Review Board (NUS-IRB Ref: N-20-053E). GEL study and MEC study (NUS-IRB Ref: 04-044; NUS-IRB Ref: 12–140 and CIRB Ref: 2001/001/C) were approved by the Institutional Review Board of the National University of Singapore and SingHealth Centralized Institutional Review Board (CIRB), and all participants gave informed consent prior to their participation. For the LCCS study, written informed consent was obtained from all patients and the study was approved by the SingHealth CIRB (CIRB Ref: 2018/2963).

### Measurement of diet

Similar semi-quantitative Food Frequency Questionnaires (FFQ) were used in all three studies. For each study, consumption frequency and standard portion size were collected, and pictures of each item portion were used during the interview. Consumption frequency was converted into average frequency per week, and the portion size was converted into the number of standard servings. The average frequency per week and number of standard servings were multiplied to obtain the number of standard servings consumed per week (Supplementary Table S1). Fresh fruit consumption was the summed weekly consumption of fresh fruits. Vegetable consumption was defined as the sum of green, leafy, and other vegetables. Fish, chicken/poultry, pork/other meat, and preserved meat intake were summed as total meat consumption. Preserved meat was summed weekly consumption of bacon, ham, luncheon meat (canned), and sausages (Supplementary Table S2). The tertile cut-off values were chosen based on the consumption among the controls. Total energy intake per week was calculated based on the energy and nutrient composition of food by the Health Promotion Board (HPB) Singapore (34) (Supplementary Table S3). To reduce information bias, we calculated the total energy intake of participants and excluded outliers to improve the robustness of our study. Outliers were defined as those with a total energy intake < 2.5th or higher than 97.5th centiles (35).

### Covariate definition

All covariates were collected in the questionnaire and adjusted in all logistic models, including sex (male vs. female), age (years, continuous), ethnicity, educational level, family history of lung cancer, smoking status, body mass index (BMI), and total energy intake (kcal, continuous). Smoking status was divided into never and ever smokers. To avoid residual confounding by smoking, ever smokers were further categorized as smoking duration <20 years, 20–40 years, and ≥40 years. Ethnicity was categorized as Chinese, Malay, Indian and others. Body mass index (BMI, kg/m^2^) was categorized as underweight (< 18.5 kg/m^2^), normal weight (18.5–24.9 kg/m^2^), overweight (25–29.9 kg/m^2^), and obese (≥ 30 kg/m^2^). Educational level was categorized as 0 years, ≤ 6 years, and >6 years of education. Family history of lung cancer was categorized as no family history of any cancers, family history of lung cancer, and family history of other cancers.

### Statistical analyses

Differences in baseline characteristics between the cases and controls were assessed using the *t*-test or Wilcoxon rank test based on the normality distribution for continuous variables, and Fisher's exact test for categorical variables. The multivariable logistic regression model was used to estimate the odds ratios (ORs) and 95% confidence intervals (CIs) of the association between meat, vegetables, and fruits consumption with lung cancer risk.

In order to reduce the potential selection bias from the age difference between cases and controls, a sensitivity analysis was conducted to match cases and controls using the propensity score nearest neighbor matching (1:1 matching by age, no replacement) (36). The matching caliper width was set as 0.2 as suggested in the previous studies (37, 38). The conditional logistic regression was performed in the sensitivity analysis for matched cases and controls to estimate the ORs and 95% CIs.

Stratification analyses by smoking status, different subtypes of lung cancer (non-small cell lung cancer, adenocarcinoma, squamous cell carcinoma), and *EGFR* status were also conducted. We also did a further subgroup analysis among non-smoking Chinese females as they are at a higher risk of *EGFR*-positive lung cancer (10, 39). All statistical tests were conducted as two-sided, and a *P*-value < 0.05 was considered as being statistically significant. All analyses were performed in Stata 16.1 (Stata Corporation, College Station, Texas, USA).

## Results

A total of 3,644 lung cancer cases and 13,964 controls were included. After excluding participants with missing information, 3,170 cases and 4,238 controls were included in the final analysis. As shown in Table 1, among cases with known *EGFR* status, *EGFR* mutation (*EGFR*+) was detected in 1,084 (57.29%) lung cancer cases, and *EGFR* wildtype (*EGFR*-) was detected in 808 cases (42.71%). Non-small cell lung cancer accounted for about 87.98% (2,789 cases) of the total lung cancer cases, of which the majority (2,242 cases, 80.39%) were adenocarcinoma. Compared with controls, lung cancer cases were significantly older and more likely to be males, have a family history of lung cancer, have lower educational levels, and lower BMI.

**Table 1:** Baseline characteristics of lung cancer cases and controls.

**Variable**	**Controls (*****N*** = **4,238)**			**Cases (*****N*** = **3,170)**		***P*** **-value ^a^**
	***wn***	**%**		***n***	**%**	
**Age at enrolment / Age at diagnosis, years**				
Mean (SD)	44.18 (15.21)			63.67 (10.99)		**<0.001**
<50	2,778	65.55		330	10.41	
50–59.9	782	18.45		740	23.34	
60–69.9	382	9.01		1,077	33.97	
≥ 70	296	6.98		1,023	32.27	
**Gender**						**<0.001**
Male	1,504	35.49		1,712	54.01	
Female	2,734	64.51		1,458	45.99	
**Ethnicity**						**<0.001**
Chinese	1,747	41.22		2,699	85.14	
Malay	1,180	27.84		207	6.53	
Indian and others ^b^	1,311	30.93		264	8.33	
**Education**						**<0.001**
0 years	328	7.74		425	13.41	
≤6 years	855	20.17		1,025	32.33	
>6 years	3,055	72.09		1,720	54.26	
**Family history of cancer (first-degree)**						
No	3,501	82.61		2,027	63.94	**<0.001**
Yes- Lung cancer	77	1.82		335	10.57	
Yes- Other cancers	660	15.57		808	25.49	
**Smoking status**					
Never smoker	2,753	64.96		1,632	51.48	**<0.001**
Ever smoker	1,485	35.04		1,538	48.52	
Smoking duration <20 years	646	15.24		160	5.05	
Smoking duration 20–40 years	381	8.99		443	13.97	
Smoking duration ≥40 years	84	1.98		831	26.21	
Unknown/missing	374	8.82		104	3.28	
**Usual body mass index, kg/m** ^**2**^						
Mean (SD)	25.54 (5.38)			22.98 (4.03)		**<0.001**
< 18.5	236	5.57		359	11.32	
18.5–24.9	1,965	46.37		1,999	63.06	
25.0–29.9	1,317	31.08		640	20.19	
≥ 30.0	720	16.99		172	5.43	
**Total energy intake of fruits, vegetables, and meat, kcal/week**						**<0.001**
Mean (SD)	2809.28 (1427.82)			2885.32 (1319.98)		
**Total fruit consumption (standard servings/week)**						**<0.001**
Mean (SD)	5.69 (5.54)			4.60 (4.18)		
**Total vegetable consumption (standard servings/week)**						**<0.001**
Mean (SD)	14.10 (12.66)			15.51 (12.58)		
**Total meat consumption (standard servings/week)**						**<0.001**
Mean (SD)	8.20 (5.22)			8.54 (4.66)		
**Lung cancer types–*****EGFR*** **status**						
*EGFR* Mutant (+)	-	-		1084	34.19	
*EGFR* Wild type (-)				808	25.49	
Unknown/not tested				1278	40.32	
**Lung cancer types–histologic types**						
Non-small cell carcinoma	-	-		2789	87.98	
Adenocarcinoma				2242	80.39	
Squamous cell carcinoma				399	14.30	
Large cell carcinoma				21	0.75	
Unspecified NSCLC				127	4.55	
Small cell lung cancer				165	5.21	
Neuroendocrine carcinoma				37	1.17	
Others ^c^				179	5.65	

SD, standard deviation; EGFR, epidermal growth factor receptor; NSCLC, non-small cell lung cancer.

a P-values were obtained using the t-test or Wilcoxon rank test for continuous variables, and Fisher's exact test for categorical variables.

b Other ethnicity included Bangladeshi, Brunei, Burmese, Cambodian, Caucasian, Eurasian, Filipino, Indonesian, Korean, Pakistani, Sri Lanka, Thai, United Arab Emirates (UAE), and Vietnamese.

c Other lung cancer types included adenocarcinoma mixed with neuroendocrine carcinoma, adenosquamous carcinoma, clinical diagnosis only, lymphoepithelioma-like carcinoma, salivary gland-type tumors, sarcomatoid carcinoma, and other unspecified lung cancer.

Bold values refer to statistically significant results with *P* < 0.05.

We found a significant inverse association between high fruit consumption (3rd tertile) and the risk of lung cancer (OR = 0.77, 95% CI = 0.65–0.91), as compared to low fruit consumption (1st tertile) (Table 2). Significant inverse associations were also observed among *EGFR*+ (OR = 077, 95% CI = 0.61–0.96) and *EGFR*- lung cancer (OR = 0.72, 95% CI = 0.55–0.94). For total vegetable consumption, as compared to low vegetable consumption (1st tertile), although the third tertile did not reach statistical significance (ORs = 0.85, 95% CI = 0.71–1.02), a significantly lower risk of lung cancer was observed among those with median consumption of vegetables, with an OR of 0.77 (95% CI = 0.65–0.91). A similar trend was observed in both *EGFR* + lung cancer and *EGFR*–lung cancer, however, the high consumption of total vegetables was statistically significant only among *EGFR*+ lung cancer (OR = 0.69, 95% CI = 0.54–0.88).

**Table 2:** Association between consumption of fruits, vegetables, and meat with risk of lung cancer subtypes.

**Amount of food intake (Standard servings per week)**	**Controls** **(*****N*** = **4,238)**		**Cases** **(*****N*** = **3,170)**		**Adjusted OR (95% CI)**	***EGFR*** + **Cases** **(*****N*** = **1,084)**		**Adjusted OR (95% CI)**	***EGFR*** −**Cases** **(*****N*** = **808)**		**Adjusted OR (95% CI)**
	***n***	***%***	***n***	***%***		***n***	***%***		***n***	**%**	
Fresh fruits ^a^											
Low (≤2.5)	1,529	36.08	1,535	48.42	1	452	41.70	1	419	51.86	1
Medium (>2.5–≤6.9)	1,236	29.16	598	18.86	**0.54 (0.46–0.65)**	191	17.62	**0.47 (0.37–0.60)**	151	18.69	**0.57 (0.43–0.74)**
High (>6.9)	1,473	34.76	1,037	32.71	**0.77 (0.65–0.91)**	441	40.68	**0.77 (0.61–0.96)**	238	29.46	**0.72 (0.55–0.94)**
Vegetables ^a^											
Low (≤7.5)	1,318	31.10	1,099	34.67	1	350	32.29	1	310	38.37	1
Medium (>7.5–≤15)	1,563	36.88	969	30.57	**0.77 (0.65–0.91)**	344	31.73	**0.70 (0.56–0.88)**	238	29.46	**0.66 (0.51–0.85)**
High (>15)	1,357	32.02	1,102	34.76	0.85 (0.71–1.02)	390	35.98	**0.69 (0.54–0.88)**	260	32.18	0.76 (0.58–1.01)
Total Meat ^a^^,^^b^											
Low (≤5)	1,326	31.29	826	26.06	1	249	22.97	1	180	22.28	1
Medium (>5–≤9)	1,470	34.69	1,121	35.36	**1.56 (1.29–1.88)**	405	37.36	**1.94 (1.51–2.50)**	300	37.13	**1.73 (1.29–2.32)**
High (>9)	1,442	34.03	1,223	38.58	**2.10 (1.58–2.79)**	430	39.67	**2.20 (1.50–3.24)**	328	40.59	**2.86 (1.84–4.47)**
Fish ^c^											
Low (≤1.75)	1,400	33.03	917	28.93	1	279	25.74	1	230	28.47	1
Medium (>1.75–≤3.5)	1,456	34.36	1,263	39.84	**1.38 (1.16–1.64)**	451	41.61	**1.66 (1.31–2.11)**	327	40.47	1.28 (0.97–1.69)
High (>3.5)	1,382	32.61	990	31.23	**1.49 (1.20–1.85)**	354	32.66	**1.90 (1.41–2.57)**	251	31.06	**1.66 (1.55–3.18)**
Chicken or Poultry ^c^											
Low (≤1.25)	1,527	36.03	1,479	46.66	1	435	40.13	1	346	42.82	1
Medium (>1.25–≤3)	1,507	35.56	1,159	36.56	1.06 (0.90–1.26)	442	40.77	**1.27 (1.01–1.60)**	314	38.86	1.09 (0.83–1.41)
High (>3)	1,204	28.41	532	16.78	0.96 (0.76–1.21)	207	19.10	**1.40 (1.01–1.94)**	148	18.32	1.14 (0.79–1.65)
Pork and other meat ^c^											
Low (≤0.5)	1,989	46.93	867	27.35	1	266	24.54	1	220	27.23	1
Medium (>0.5–≤1.25)	905	21.35	655	20.66	0.84 (0.70–1.02)	217	20.02	1.07 (0.82–1.39)	136	16.83	0.80 (0.58–1.09)
High (>1.25)	1,344	31.71	1,648	51.99	**1.32 (1.08–1.62)**	601	55.44	**1.94 (1.46–2.56)**	452	55.94	**1.36 (1.00–1.89)**
Preserved meat ^c^^,^^d^											
Non-consumer	2,437	57.50	1,200	37.85	1	364	33.58	1	257	31.81	1
Low (≤1)	1,030	24.30	1,333	42.05	**3.40 (2.88–4.01)**	515	47.51	**5.28 (4.23–6.59)**	384	47.52	**5.66 (4.34–7.36)**
High (>1)	771	18.19	637	20.09	**3.02 (2.46–3.70)**	205	18.91	**3.49 (2.63–4.65)**	167	20.67	**4.00 (2.89–5.54)**

OR, odds ratio; CI, confidence interval; EGFR, epidermal growth factor receptor.

a Adjusted for age, gender, education, ethnicity, BMI, smoking status and duration, family history of lung cancer, total energy intake, fruit, vegetable, and meat consumption.

b Summed weekly consumption of fish, chicken or poultry, pork and other meat, and preserved meat.

c Adjusted for age, gender, education, ethnicity, BMI, smoking status and duration, family history of lung cancer, total energy intake, fruit, vegetable, and fish, chicken or poultry, pork and other meat, and preserved meat consumption.

d As a large number of participants did not consume preserved meat, it was divided into non-consumer, consumed ≤1 standard serving, and consumed >1 standard serving per week.

Bold values refer to statistically significant results with *P* < 0.05.

Overall, positive associations between total meat intake and lung cancer were reported in our study population. Compared with low meat consumption (1st tertile), a statistically significant positive association between higher consumption (3rd tertile) of total meat and the elevated risk of lung cancer was observed (OR = 2.10, 95% CI = 1.58–2.79). When the analysis was stratified by *EGFR* status, statistically significant positive associations were also found for *EGFR*+ lung cancer (OR = 2.20, 95% CI = 1.50–3.24) and *EGFR*- lung cancer (OR = 2.86, 95% CI = 1.84–4.47). In addition, we observed positive associations between higher consumption of fish (OR = 1.49, 95% CI = 1.20–1.85), pork and other meats (OR = 1.32, 95% CI = 1.08–1.62), preserved meat (OR = 3.02, 95% CI = 2.46–3.70), with the risk of lung cancer.

When stratified by different subtypes of lung cancer, we observed similar associations among non-small cell lung cancer, adenocarcinoma, and squamous cell carcinoma (Table 3). Higher fruit consumption was significantly and inversely associated with the risk of all subtypes of lung cancer. A statistically significant positive association between higher consumption of total meat and the elevated risk of non-small cell lung cancer (OR = 1.99, 95% CI = 1.49–2.67), and adenocarcinoma (OR = 2.06, 95% CI = 1.52–2.79) were observed, except for squamous cell carcinoma (OR = 1.21, 95% CI = 0.59–2.51).

**Table 3:** Association between consumption of fruits, vegetables, and meat with risk of different histologic types of lung cancer.

**Amount of food intake (Standard servings per week)**	**NSCLC (*****N*** = **2,789)**		**Adjusted OR (95% CI)^a^**	**Adenocarcinoma** **(*****N*** = **2,242)**		**Adjusted OR (95% CI)^a^**	**Squamous cell carcinoma** **(*****N*** = **399)**		**Adjusted OR (95% CI)^a^**
	***n***	***%***		***n***	***%***		***n***	***%***	
Fresh fruits ^a^									
Low (≤2.5)	1,324	47.47	1	997	44.47	1	246	61.65	1
Medium (>2.5–≤6.9)	533	19.11	**0.56 (0.47–0.67)**	432	19.27	**0.56 (0.47–0.68)**	71	17.79	**0.58 (0.37–0.89)**
High (>6.9)	932	33.42	**0.78 (0.66–0.93)**	813	36.26	**0.80 (0.67–0.96)**	82	20.55	**0.63 (0.40–0.98)**
Vegetables ^a^									
Low (≤7.5)	969	34.74	1	743	33.14	1	182	45.61	1
Medium (>7.5–≤15)	851	30.51	**0.74 (0.62–0.88)**	690	30.78	**0.74 (0.62–0.89)**	115	28.82	**0.55 (0.37–0.83)**
High (>15)	969	34.74	**0.83 (0.69–0.99)**	809	36.08	**0.82 (0.67–0.99)**	102	25.56	0.66 (0.41–1.05)
Total Meat ^a^^,^ ^b^									
Low (≤5)	717	25.71	1	557	24.84	1	119	29.82	1
Medium (>5–≤9)	992	35.57	**1.51 (1.24–1.83)**	801	35.73	**1.56 (1.27–1.90)**	140	35.09	1.09 (0.68–1.74)
High (>9)	1,080	38.72	**1.99 (1.49–2.67)**	884	39.43	**2.06 (1.52–2.79)**	140	35.09	1.21 (0.59–2.51)
Fish ^c^									
Low (≤1.75)	797	28.58	1	621	27.70	1	131	32.83	1
Medium (>1.75–≤3.5)	1,116	40.01	**1.36 (1.14–1.63)**	887	39.56	**1.38 (1.15–1.66)**	171	42.86	1.43 (0.92–2.23)
High (>3.5)	876	31.41	**1.48 (1.18–1.85)**	734	32.74	**1.55 (1.23–1.95)**	97	24.31	1.10 (0.60–2.01)
Chicken or Poultry ^c^									
Low (≤1.25)	1,281	45.93	1	1,007	44.92	1	200	50.13	1
Medium (>1.25–≤3)	1,040	37.29	1.08 (0.91–1.29)	837	37.33	1.06 (0.89–1.27)	145	36.34	1.08 (0.70–1.67)
High (>3)	468	16.78	0.95 (0.75–1.21)	398	17.75	0.97 (0.75–1.24)	54	13.53	1.29 (0.68–2.42)
Pork and other meat ^c^									
Low (≤0.5)	764	27.39	1	624	27.83	1	96	24.06	1
Medium (>0.5–≤1.25)	567	20.33	0.83 (0.68–1.01)	451	20.12	0.83 (0.68–1.02)	79	19.80	0.82 (0.49–1.36)
High (>1.25)	1,458	52.28	**1.24 (1.01–1.54)**	1,167	52.05	**1.26 (1.01–1.57)**	224	56.14	1.23 (0.73–2.10)
Preserved meat ^c^^,^ ^d^									
Non-consumer	1,040	37.29	1	828	36.93	1	140	35.09	1
Low (≤1)	1,182	42.38	**3.54 (2.98–4.20)**	968	43.18	**3.77 (3.16–4.50)**	174	43.61	**3.40 (2.16–5.34)**
High (>1)	567	20.33	**3.07 (2.48–3.79)**	446	19.89	**3.13 (2.51–3.90)**	85	21.30	**4.18 (2.50–7.00)**

OR, odds ratio; CI, confidence interval; NSCLC, non-small cell lung cancer.

a Adjusted for age, gender, education, ethnicity, BMI, smoking status and duration, family history of lung cancer, total energy intake, fruit, vegetable, and meat consumption.

b Summed weekly consumption of fish, chicken or poultry, pork and other meat, and preserved meat.

c Adjusted for age, gender, education, ethnicity, BMI, smoking status and duration, family history of lung cancer, total energy intake, fruit, vegetable, and fish, chicken or poultry, pork and other meat, and preserved meat consumption.

d As a large number of participants did not consume preserved meat, it was divided into non-consumer, consumed ≤ 1 standard serving, and consumed >1 standard serving per week.

Bold values refer to statistically significant results with *P* < 0.05.

Among never smokers, as compared to the lowest tertile, the highest total meat consumption group was associated with a higher risk of lung cancer across all strata of never smokers (never smokers, never-smoking females, and never-smoking Chinese females) (Table 4). No statistically significant associations between total vegetable consumption and risk of lung cancer were observed. For the age-matched sensitivity analyses 2,340 cases were age-matched with 2,340 controls. A total of 1,084 EGFR+ and 909 EGFR- cases were also age-matched with the same number of controls, respectively. Overall, the results were similar with the main analyses (Supplementary Tables S4–S6). Compared with low fruit consumption, a significant inverse association between high fruit consumption and the risk of lung cancer remained (OR = 0.79, 95% CI = 0.64–0.98). The statistically significant positive association between higher consumption (3rd tertile) of total meat and the elevated risk of lung cancer was also observed (OR = 1.92, 95% CI = 1.34–2.75), as compared with low meat consumption (1st tertile).

**Table 4:** Association between consumption of fruits, vegetables, and meat with risk of lung cancer among non-smokers.

**Amount of food intake (Standard servings per week)**	**Controls**		**Cases**		**Adjusted Odds ratio (95% CI)**	***EGFR*** + **Cases**		**Adjusted OR (95% CI)^a^**	***EGFR*** **– Cases**		**Adjusted OR (95% CI)^a^**
	***n***	***%***	***n***	**%**		***n***	***%***		***n***	**%**	
**Never-smokers**	N = 2,753		N = 1,632			N = 841			N = 305		
Fresh fruit ^a^											
Low (≤2.5)	855	31.06	631	38.66	1	328	39.00	1	122	40.00	1
Medium (>2.5–≤6.9)	818	29.71	337	20.65	**0.56 (0.47–0.69)**	152	18.07	**0.48 (0.37–0.64)**	63	20.66	**0.57 (0.39–0.82)**
High (>6.9)	1,080	39.23	664	40.69	**0.80 (0.66–0.98)**	361	42.93	**0.78 (0.61–0.99)**	120	39.34	0.80 (0.57–1.13)
Vegetables ^a^											
Low (≤8.5)	916	33.27	539	33.03	1	279	33.17	1	104	34.10	1
Medium (>8.5–≤16.5)	949	34.47	489	29.96	0.85 (0.70–1.05)	257	30.56	0.85 (0.66–1.09)	88	28.85	**0.68 (0.48–0.96)**
High (>16.5)	888	32.26	604	37.01	1.01 (0.82–1.26)	305	36.27	0.90 (0.68–1.18)	113	37.05	0.89 (0.62–1.29)
Total Meat ^b^											
Low (≤5)	954	34.65	443	27.14	1	204	24.26	1	75	24.59	1
Medium (>5–≤9)	918	33.35	593	36.34	**1.95 (1.57–2.43)**	315	37.46	**1.75 (1.29–2.38)**	106	34.75	**2.39 (1.62–3.52)**
High (>9)	881	32.00	596	36.52	**2.64 (1.88–3.70)**	322	38.29	1.09 (0.69–1.71)	124	40.66	**4.56 (2.55–8.15)**
**Never-smoking females**	*N* = 2234	N = 1221		*N* = 600		*N* = 217	
Fresh fruit											
Low (≤2.5)	708	31.69	468	38.33	1	225	37.50	1	88	40.55	1
Medium (>2.5–≤6.9)	654	29.27	271	22.19	**0.60 (0.48–0.75)**	113	18.83	**0.50 (0.37–0.68)**	51	23.50	**0.66 (0.44–0.98)**
High (>6.9)	872	39.03	482	39.48	**0.80 (0.64–0.99)**	262	43.67	0.83 (0.63–1.10)	78	35.94	0.76 (0.51–1.13)
Vegetables											
Low (≤8.5)	747	33.44	403	33.01	1	194	32.33	1	79	36.41	1
Medium (>8.5–≤16.5)	754	33.75	362	29.65	0.81 (0.65–1.01)	185	30.83	0.82 (0.62–1.08)	56	25.81	**0.57 (0.38–0.84)**
High (>16.5)	733	32.81	456	37.35	0.99 (0.78–1.25)	221	36.83	0.87 (0.65–1.18)	82	37.79	0.88 (0.58–1.32)
Total Meat ^b^											
Low (≤5)	813	36.39	374	30.63	1	167	27.83	1	61	28.11	1
Medium (>5–≤9)	739	33.08	450	36.86	**1.92 (1.52–2.44)**	228	38.00	**2.18 (1.61–2.95)**	75	34.56	**2.42 (1.58–3.72)**
High (>9)	682	30.53	397	32.51	**2.39 (1.66–3.45)**	205	34.17	**2.46 (1.53–3.94)**	81	37.33	**5.06 (2.64–9.72)**
**Never-smoking Chinese females**	*N* = 1112	*N* = 1081		*N* = 520		*N* = 171	
Fresh fruit											
Low (≤2.75)	372	33.79	428	40.34	1	196	38.06	1	76	45.24	1
Medium (>2.75–≤6.9)	283	25.70	216	20.36	0.80 (0.62–1.02)	93	18.06	0.77 (0.56–1.06)	33	19.64	0.79 (0.49–1.25)
High (>6.9)	446	40.51	417	39.30	1.13 (0.90–1.42)	226	43.88	1.31 (0.98–1.73)	59	35.12	1.09 (0.71–1.66)
Vegetables											
Low (≤10)	365	33.15	397	37.42	1	182	35.34	1	70	41.67	1
Medium (>10–≤19)	377	34.24	349	32.89	0.97 (0.77–1.22)	189	36.70	1.08 (0.81–1.44)	49	29.17	0.77 (0.50–1.17)
High (>19)	359	32.61	315	29.69	1.11 (0.85–1.43)	144	27.96	1.03 (0.74–1.43)	49	29.17	1.08 (0.68–1.73)
Total Meat ^b^											
Low (≤5.25)	371	33.70	336	31.67	1	142	27.57	1	45	26.79	1
Medium (>5.25–≤10)	398	36.15	457	43.07	**2.54 (1.95–3.31)**	228	44.27	**3.14 (2.23–4.43)**	73	43.45	**4.24 (2.51–7.15)**
High (>10)	332	30.15	268	25.26	**4.64 (2.95–7.30)**	145	28.16	**6.33 (3.56–11.25)**	50	29.76	**15.72 (6.46–38.26)**

OR, odds ratio; NSCLC, non-small cell lung cancer.

a Adjusted for age, gender, education, ethnicity, BMI, smoking status and duration, family history of lung cancer, total energy intake, fruit, vegetable, and meat consumption.

b Summed weekly consumption of fish, chicken/poultry, pork/meat, and preserved meat.

Bold values refer to statistically significant results with P-value < 0.05.

## Discussion

In this study, we assessed the association between dietary factors and the risk of different histological and molecular subtypes of lung cancer. After adjusting for covariates, we identified higher consumption of total fresh fruits associated with a lower risk of lung cancer. In contrast, the higher total meat consumption, fish, pork, and preserved meat were statistically associated with elevated lung cancer risk. A significant inverse association between higher vegetable consumption and risk of *EGFR*+ lung cancer was identified, however, this association was not statistically significant among *EGFR*- lung cancer.

Consistent with previous studies, our findings showed that higher fruit consumption was correlated with a lower risk of lung cancer (40, 41). However, we did not find the monotonic decreasing ORs when comparing medium and higher fruit consumption groups. This may be attributed to the non-linear association reported in the previous study: lung cancer risk decreased for fruit consumption up to 200–300 grams per day, and no further decrease for higher consumption (42, 43). Compared with vegetables, we observed a pronounced association between fruits consumptions and lung cancer across all subtypes of lung cancer and among all subgroup populations. According to several previous studies, this pronounced protective evidence of fruits was repeatedly reported, however, the potential mechanisms still need to be investigated (41, 44, 45).

For vegetable consumption, our findings concur with previous work that an inverse association among higher consumption groups was reported (46), although we did not find a clear dose-response relationship. Similarly, a recent literature review by the World Cancer Research Fund supported the non-linear relationship between vegetable consumption and the risk of lung cancer, with decreasing risks for 300–400 grams per day and no further decrease for higher intake levels (42, 43). When stratified by smoking status, we did not find any significant associations among never-smokers, never-smoking females, or never-smoking Chinese females. Vieira et al. (13) and Smith-Warner et al. (47) also demonstrated that this protective effect was only significant among current smokers but was not statistically significant among former and never smokers. Interestingly, in the stratified analysis by *EGFR* status, a significantly decreased lung cancer risk was found only among *EGFR*+ lung cancer. Hamaguchi et al. reported that an alkaline diet (more vegetables and fruits, and less meat and dairy products) enhanced the effect of *EGFR*-TK inhibitor treatment in lung cancer patients with *EGFR* mutations (22). Our results may provide some insights into the potential mechanisms. Furthermore, the curcumin from turmeric (48, 49), Lupeol (a kind of phytosterol derived from fruits and vegetables) (50), and procyanidins-rich diets (51) have been shown to inhibit *EGFR* activation and have anti-cancer effects in lung cancer in multiple steps. However, we noted that the 95% CIs of the estimates in association for vegetable consumption and lung cancer by *EGFR* status were largely overlapping, i.e., 0.69 (0.54–0.88) and 0.76 (0.58–1.01). In the sensitivity analysis, no significant associations between vegetable consumption and *EGFR* +/- lung cancer were found. Therefore, this difference may be due to chance.

We found a significant positive association between total meat consumption and the risk of lung cancer after adjusting for covariates and total energy intake. Similar to our findings, a dose-response association was also found by Xue et al. with every increase of 120 g per day of red meat consumption, the risk of lung cancer increased by 35% (RR = 1.35, 95%CI = 1.25–1.46) (14). Lam et al. reported a significant positive association between higher meat intake and the risk of lung adenocarcinoma and squamous cell carcinoma (17). Similarly, our results also support the statistically significant relationship between higher meat intake and the risk of lung adenocarcinoma. As for the lack of statistically significant results for squamous cell carcinoma, this may be due to the limited number of cases in this group.

In this study, we were able to assess the effect of the consumption of total fruits, vegetables, and meat on the risk of lung cancer by specific subtypes. Based on our results, we did not observe any huge differences between different lung cancer subtypes. Higher consumption of fruits and vegetables was less pronounced among adenocarcinoma cases as compared to squamous cell carcinoma cases. Few studies have analyzed the effect of fruits and vegetables among specific lung cancer subtypes, and the results were inconsistent: four previous studies demonstrated statistically insignificant associations for small-cell carcinoma, adenocarcinoma, squamous cell carcinoma, and large cell lung carcinoma (13, 14, 40, 52); whereas Voorrips et al. revealed a weaker protective effect for adenocarcinomas than for other types of tumors, which was consistent with our results (53).

Some potential mechanisms have been proposed but the conclusions from different studies remained inconsistent. The protective effect of fruits and vegetables was attributed to biologically active compounds, including flavonoids and carotenoids (54, 55). Flavonoids found in fruit modular cytochrome P450 enzyme systems are involved in the metabolism of carcinogens (56). However, another study indicated that the intake of carotene supplementation was not associated with a decreasing risk of lung cancer (57). Besides, the protective effect may likely result from a combination of each constituent in influences several pathways involved in lung carcinogenesis (43). Red meat and processed meat are sources of saturated fats and heme iron, and several mutagens when cooked at a high temperature, including polycyclic aromatic hydrocarbons (PHAs) and heterocyclic amines (HCAs). These chemicals and mutagens may contribute to an increased risk of lung cancer (16, 58–60). However, a cohort study demonstrated a non-significant association between cooking methods, intake of specific meat mutagens or heme iron, and the risk of lung cancer (19). Therefore, further studies may be needed to characterize the mechanisms in these associations.

Although the results of this study support the hypothesis that fruit consumption is inversely associated with the risk of lung cancer and the consumption of meat is positively associated with the risk of lung cancer, there are several caveats to consider. Firstly, the cases and controls were taken from three different studies, which were carried out over different periods and used different questionnaires. Consumption of fruits, vegetables, and meat was collected in different ways; interviewers might have been trained differently, eliciting different responses from subjects. Although we have tried our best to combine those datasets appropriately and harmonize the variables, these limitations may affect the robustness of our findings, which may attenuate the results. The three studies enrolled subjects from different time periods, although all the cohorts started recruitment in 2005–2007. To control for the potential effects of the different enrollment time periods, we adjusted for the enrollment period in our model, and we found that the overall ORs and 95% CIs remained similar. In addition, in these recent 10 years, although Singaporean diet format and categories did not change much, we cannot deny that some participants may tend to eat healthily or improve their diet quality during this time (61, 62). Based on the Singapore National Nutrition Survey Report, from 2004 to 2018, the average daily intake increased a bit, from 2290 to 2470 kcal per day. The consumption of fruits and vegetables increased, but the percentage of protein remained stable at 14–15% of the total energy (63, 64). Overall, as more than half of our controls were recruited after 2008, this difference in the recruitment period may slightly overestimate our current results.

Secondly, recall bias is a major limitation for case-control studies. Although the food frequency questionnaire has been used previously and showed validity (18, 65), the participants may underreport or overreport some specific food items when asked to recall their past diet. Cases, especially among females and non-smokers, may be more likely than controls to report unhealthy diet habits and vice versa. We have made efforts to minimize this limitation by training the interviewers to limit investigator bias. Further research in a prospective cohort study is warranted to validate our findings.

Thirdly, we were unable to access the relative importance of each constituent and the effect of other food items, such as flavonoids, carotenoids rice, eggs, fast foods, soy, and dairy products due to the questionnaires. Furthermore, because the questionnaires of LCCS and MEC datasets were limited to each fruit and vegetable item portion size and frequency, we can only use the average energy intake to present each category to calculate the total energy intake (including fruits, green leafy, or other vegetables, and each kind of meat). Therefore, there are likely to be measurement errors. To avoid an under- or over-estimation of total energy intake, a total energy intake of < 2.5th or higher than 97.5th percentiles was excluded. Despite the limitations, to our knowledge, this is the first study of dietary factors and the risk of lung cancer by *EGFR* +/- and histologic subtypes among Southeast Asians.

## Conclusions

In summary, we found that higher vegetable consumption was significantly associated with a decreased risk of *EGFR*+ lung cancer. Consistent with prior studies, an inverse association between higher fruit consumption and lung cancer, and a positive association between higher meat consumption and lung cancer were identified. Both associations remained significant when stratified by different molecular and histological types of lung cancer. Further prospective studies are warranted to assess this association and characterize the underlying biological mechanisms.

## Data availability statement

The data analyzed in this study is subject to the following licenses/restrictions: the data are not publicly available due to privacy or ethical restrictions. Requests to access these datasets should be directed to WS, ephswj@nus.edu.sg.

## Ethics statement

This current study of using three datasets was approved by the National University of Singapore Institutional Review Board (NUS-IRB Ref: N-20-053E). The patients/participants provided their written informed consent to participate in this study.

## Author contributions

XY: conceptualization, formal analysis, and writing–original draft. GL and DT: writing–review and editing. AS: resources and writing–review and editing. DL and WS: writing–review and editing and supervision. All authors contributed to the article and approved the submitted version.

## Conflict of interest

The authors declare that the research was conducted in the absence of any commercial or financial relationships that could be construed as a potential conflict of interest.

## Publisher's note

All claims expressed in this article are solely those of the authors and do not necessarily represent those of their affiliated organizations, or those of the publisher, the editors and the reviewers. Any product that may be evaluated in this article, or claim that may be made by its manufacturer, is not guaranteed or endorsed by the publisher.
