# Supplementary material for: Dietary factors and the risk of lung cancer by epidermal growth factor receptor mutation status and histological subtypes
Source: Front Public Health. 2022 Dec 2;10:1079543. doi: 10.3389/fpubh.2022.1079543 (PMC9755194; doi:10.3389/fpubh.2022.1079543)
Supplement: Supplementary file 1 [file Table_1.DOCX]

Supplementary Material

**Supplementary Table 1. Conversion of consumption frequency to frequency per week and portion size to the number of standard servings**

|  | **Average frequency per week** | | | | |
| --- | --- | --- | --- | --- | --- |
|  | **GEL** | | **LCCS** | | **MEC** |
| **Consumption frequency** | **Meat** | **Fruits and vegetables** | **Meat** | **Fruits and vegetables** |  |
| Never or less than once a year/Rarely/Never | 0 | 0^a^ | 0 | 0 | 0 |
| At least once a year but less than once a month | 0.125 | 0^a^ | 0.125 | 0 | 0 |
| 1-3 times a month | 0.46 | 0.46 | 0.46 | 0.46 | n/month*0.25 |
| Once a week | 1 | 1 | 1 | 1 | 1 |
| 2-3 times a week | 2.5 | 2.5 | 2.5 | 2.5 | n/week*1 |
| 4-6 times a week | 5 | 5 | 5 | 5 | n/week*1 |
| Once a day | 7 | 7 | 7 | 7 | 7 |
| 2-3 times a day | 17.5 | 17.5 | 17.5 | 17.5 | n/day*7 |
| More than 2-3 times a day | - | - | - | - | n/day*7 |
| **Portion size** | **Standard servings** | | | | |
| Small | 0.5 | 0.5 | 0.5 | 0.5 | - |
| Medium | 1 | 1 | 1 | 1 | 1 |
| Large | 2 | 2 | 2 | 2 | - |
| Small + Large | - | - | 2^b^ | 2^b^ | - |
| Medium + Large | - | - | 2^b^ | 2^b^ | - |

GEL, Genes and Environment in Lung Cancer study; LCCS, Lung Cancer Consortium Singapore study; MEC, Multi-ethnic Cohort study; n, number of frequency.

^a^ The GEL questionnaire did not differentiate between “Never or less than once a year” and “At least once a year but less than once a month” for fruit and vegetable intake. Therefore, to standardize between the 3 questionnaires, consumption was also assumed to be 0 for “At least once a year but less than once a month”.

^b^ The LCCS questionnaire had categories of “Small + Large” and “Medium + Large” for meat intake. As the MEC/GEL questionnaire did not have such categories. These categories were assumed to be the same as “Large” in the LCCS study.

**Supplementary Table 2. Harmonization of fruits, vegetables, and meat consumption across the three studies**

|  | **GEL** | **LCCS** | **MEC** |
| --- | --- | --- | --- |
| **Fruits** | Papaya  Apple  Orange or mandarin orange  Mango  Watermelon | Fresh fruits: Apple, orange, or mandarin orange | Orange/red/yellow fresh fruits and fruit juices: watermelon, papaya, mango, persimmon  Other fresh fruits and fruit juices: pear, apple, grape, dragon fruit |
| **Vegetables** | wong-nga-pak, Chinese cabbage, Chinese kale, head cabbage, cauliflower, kai choy, choy sum, watercress, broccoli, water convolvulus, spinach, Chinese lettuce,  tomatoes, French beans, string (long) beans, snow peas, ladies’ fingers, and carrot (red) | Cruciferous vegetables (wong-nga-pak, Chinese cabbage, Chinese kale, head cabbage, cauliflower, kai choy, choy sum, watercress, and broccoli)  Other leafy vegetables (water convolvulus, spinach, and Chinese lettuce)  Other vegetables (tomatoes, french beans, string (long) beans, snow peas, ladies’ fingers, and carrot (red)) | Pale green leafy vegetables (cabbage, Chinese cabbage, lettuce, beansprouts, cauliflower, etc)  Dark green leafy vegetables (spinach, Chinese kale, chye sim, kangkong broccoli, etc)  Tomatoes, carrots, red/yellow pepper  Legumes/pulses (beans, peas)  Mixed vegetables |
| **Meat** | Fish  Chicken  Pork  Preserved meat: bacon, ham, luncheon meat, sausage | Fish  Chicken  Pork  Preserved meat: bacon, ham, luncheon meat, sausage | Fish  Poultry  Meat: lean, lean and fat  Preserved meat: bacon, ham, luncheon meat, sausage |

GEL, Genes and Environment in Lung Cancer study; LCCS, Lung Cancer Consortium Singapore study; MEC, Multi-ethnic Cohort study.

**Supplementary Table 3. Conversion of the total energy intake**

|  | Items | Standard serving | Energy | Average energy ^a^ |
| --- | --- | --- | --- | --- |
| Fruits |  |  |  | 67.97 kcal |
|  | Papaya | Slice (225g) | 76.43 kcal |  |
|  | Apple, unspecified type, red, raw, with skin | Whole (150g) | 86.13 kcal |  |
|  | Mandarin orange | Whole (120g) | 52.80 kcal |  |
|  | Orange | Whole (150g) | 62.81 kcal |  |
|  | Mango, raw | ½ Whole (116g) | 80.04 kcal |  |
|  | Watermelon | Slice (119g) | 44.08 kcal |  |
|  | Dragon fruit | ½ Whole (150g) | 73.50 kcal |  |
| Vegetables |  |  |  |  |
| Green and leafy vegetables |  |  |  | 21.60 kcal |
|  | Cabbage, white, chinese, boiled, drained | Cup (170g) | 20.40 kcal |  |
|  | Kale, Chinese, raw | Stalk (41g) | 17.63 kcal |  |
|  | Lettuce, raw | Stalk (82g) | 13.91 kcal |  |
|  | Cabbage, common, raw | Cup (70g) | 15.39 kcal |  |
|  | Cauliflower, boiled, drained | Cup (124g) | 28.52 kcal |  |
|  | Watercress, raw | Cup (34g) | 6.70 kcal |  |
|  | Cucumber, raw | Whole (270g) | 43.24 kcal |  |
|  | Broccoli, boiled | Half Cup (79.8g) | 22.34 kcal |  |
|  | Spinach, Chinese, raw | Cup (56g) | 26.23 kcal |  |
| Other vegetables |  |  |  | 38.05 kcal |
|  | Tomato, raw | Whole (55g) | 12.10 kcal |  |
|  | Beans, french, raw | Cup (82g) | 23.94 kcal |  |
|  | Long beans, raw | Cup (118g) | 35.54 kcal |  |
|  | Peas, garden, fresh | Cup (157g) | 78.50 kcal |  |
|  | Snow peas, raw | Cup (100g) | 27.27 kcal |  |
|  | Carrot, mature, raw, peeled | Whole (162.37g) | 51.28 kcal |  |
|  | Peppers, capsicum, yellow | Whole (medium) (160g) | 35.20 kcal |  |
|  | Peppers, capsicum, red, raw | Whole (medium) (160g) | 40.58 kcal |  |
| Meat |  |  |  |  |
| Fish | Fried fish, unspecified | Whole (100g) | 232 kcal | 218.68 kcal |
|  | Steamed fish, unspecified | Piece (185g) | 205.35 kcal |  |
| Chicken | Chicken, drumstick, raw, lean and skin | Whole (115g) | 177.45 kcal | 267.77 kcal |
|  | Chicken, breast, baked, lean and skin | Whole (120g) | 261.85 kcal |  |
|  | Chicken, breast, battered, fried, lean and skin | Whole (140g) | 364 kcal |  |
| Pork | Pork, butterfly steak, grilled, lean and fat | Whole (120g) | 312.91 kcal | 342.60 kcal |
|  | Pork, leg, baked, lean and fat | Whole (110g) | 372.28 kcal |  |
| Preserved meat: bacon, ham, luncheon meat, sausage | Bacon, cooked, pan-fried | Slice (7.9g) | 42.11 kcal | 120.73 kcal |
|  | Ham, chicken | Slice (21g) | 30.03 kcal |  |
|  | Canned luncheon meat | Slice (46.7g) | 146.17 kcal |  |
|  | Sausage, pork, cured, smoke, linked, grilled | Piece (68g) | 264.59 kcal |  |

^a^ Because LCCS and MEC study did not collect food items separately, we calculated the total energy intake by using average energy.

**Supplementary Table 4. Association between consumption of fruits, vegetables, and meat with risk of lung cancer (age matched with caliper 0.2)**

| **Amount of food intake (Standard servings per week)** | **Controls**  **(N = 2,340)** | | **Cases**  **(N = 2,340)** | | **Adjusted OR (95% CI)** |
| --- | --- | --- | --- | --- | --- |
|  | **n** | **%** | **n** | **%** |  |
| Fresh fruits ^a^ |  |  |  |  |  |
| Low (≤2.75) | 793 | 33.89 | 1,131 | 48.33 | 1 |
| Medium (>2.75 - ≤6.75) | 649 | 27.74 | 431 | 18.42 | **0.56 (0.45 – 0.70)** |
| High (>6.75) | 898 | 38.38 | 778 | 33.25 | **0.79 (0.64 – 0.98)** |
| Vegetables ^a^ |  |  |  |  |  |
| Low (≤8) | 724 | 30.94 | 857 | 36.62 | 1 |
| Medium (>8 - ≤16) | 875 | 37.39 | 688 | 29.40 | **0.72 (0.59 – 0.89)** |
| High (>16) | 741 | 31.67 | 795 | 33.97 | 1.04 (0.83 – 1.31) |
| Total Meat ^a,b^ |  |  |  |  |  |
| Low (≤5) | 831 | 35.51 | 597 | 25.51 | 1 |
| Medium (>5 - ≤8.75) | 764 | 32.65 | 814 | 34.79 | **1.48 (1.18 – 1.88)** |
| High (>8.75) | 745 | 31.84 | 929 | 39.70 | **1.92 (1.34 – 2.75)** |
| Fish ^c^ |  |  |  |  |  |
| Low (≤2) | 892 | 38.12 | 686 | 29.32 | 1 |
| Medium (>2 - ≤4) | 781 | 33.38 | 925 | 39.53 | **1.97 (1.57 – 2.48)** |
| High (>4) | 667 | 28.50 | 729 | 31.15 | **2.82 (2.10 – 3.81)** |
| Chicken or Poultry ^c^ |  |  |  |  |  |
| Low (≤1) | 844 | 36.07 | 859 | 36.71 | 1 |
| Medium (>1 - ≤2.5) | 849 | 36.28 | 1,090 | 46.58 | **1.31 (1.06 – 1.61)** |
| High (>2.5) | 647 | 27.65 | 391 | 16.71 | 0.75 (0.56 – 1.01) |
| Pork and other meat ^c^ |  |  |  |  |  |
| Low (≤0.25) | 839 | 35.85 | 441 | 18.85 | 1 |
| Medium (>0.25 - ≤1.25) | 834 | 35.64 | 717 | 30.64 | 0.82 (0.65 – 1.04) |
| High (>1.25) | 667 | 28.50 | 1,182 | 50.51 | **1.37 (1.02 – 1.84)** |
| Preserved meat ^c,d^ |  |  |  |  |  |
| Non-consumer | 1,651 | 70.56 | 900 | 38.46 | 1 |
| Low (≤1) | 449 | 19.19 | 961 | 41.07 | **2.59 (2.09 – 3.21)** |
| High (>1) | 240 | 10.26 | 479 | 20.47 | **2.65 (2.00 – 3.50)** |

Abbreviations: OR, odds ratio; CI, confidence interval.

^a^ Adjusted for gender, education, ethnicity, BMI, smoking status, family history of lung cancer, total energy intake, fruit, vegetable, and meat consumption.

^b^ Summed weekly consumption of fish, chicken or poultry, pork and other meat, and preserved meat.

^c^ Adjusted for gender, education, ethnicity, BMI, smoking status, family history of lung cancer, total energy intake, fruit, vegetable, and fish, chicken or poultry, pork and other meat, and preserved meat consumption.

^d^ As a large number of participants did not consume preserved meat, it was divided into non-consumer, consumed ≤1 standard serving, and consumed >1 standard serving per week.

**Supplementary Table 5. Association between consumption of fruits, vegetables, and meat with risk of *EGFR*+ lung cancer subtypes (age matched with caliper 0.2)**

| **Amount of food intake (Standard servings per week)** | **Controls**  **(N = 1084)** | | ***EGFR*+ Cases**  **(N = 1084)** | | **Adjusted OR (95% CI)** |
| --- | --- | --- | --- | --- | --- |
|  | **n** | **%** | **n** | **%** |  |
| Fresh fruits ^a^ |  |  |  |  |  |
| Low (≤2.5) | 347 | 32.01 | 452 | 41.70 | 1 |
| Medium (>2.5 - ≤6.9) | 311 | 28.69 | 191 | 17.62 | **0.36 (0.26 – 0.51)** |
| High (>6.9) | 426 | 39.30 | 441 | 40.68 | **0.65 (0.49 – 0.86)** |
| Vegetables ^a^ |  |  |  |  |  |
| Low (≤8.75) | 352 | 32.47 | 380 | 35.06 | 1 |
| Medium (>8.75 - ≤17) | 368 | 33.95 | 370 | 34.13 | 0.92 (0.67 – 1.25) |
| High (>17) | 364 | 33.58 | 334 | 30.81 | 0.84 (0.61 – 1.16) |
| Total Meat ^a,b^ |  |  |  |  |  |
| Low (≤4.75) | 372 | 34.32 | 207 | 19.10 | 1 |
| Medium (>4.75 - ≤8.75) | 349 | 32.20 | 439 | 40.50 | **2.63 (1.88 – 3.68)** |
| High (>8.75) | 363 | 33.49 | 438 | 40.41 | **3.38 (2.02 – 5.68)** |
| Fish ^c^ |  |  |  |  |  |
| Low (≤2) | 385 | 35.52 | 280 | 25.83 | 1 |
| Medium (>2 - ≤4) | 356 | 32.84 | 450 | 41.51 | **2.33 (1.61 – 3.39)** |
| High (>4) | 343 | 31.64 | 354 | 32.66 | **5.30 (3.24 – 8.65)** |
| Chicken or Poultry ^c^ |  |  |  |  |  |
| Low (≤0.5) | 355 | 32.75 | 173 | 15.96 | 1 |
| Medium (>0.5 - ≤2.5) | 502 | 46.31 | 704 | 64.94 | **2.07 (1.42 – 3.00)** |
| High (>2.5) | 227 | 20.94 | 207 | 19.10 | **2.25 (1.33 – 3.82)** |
| Pork and other meat ^c^ |  |  |  |  |  |
| Low (≤0.46) | 360 | 33.21 | 170 | 15.68 | 1 |
| Medium (>0.46 - ≤1.5) | 436 | 40.22 | 313 | 28.87 | 0.79 (0.53 – 1.18) |
| High (>1.5) | 288 | 26.57 | 601 | 55.44 | **3.03 (1.84 – 5.00)** |
| Preserved meat ^c,d^ |  |  |  |  |  |
| Non-consumer | 851 | 78.51 | 364 | 33.58 | 1 |
| Consumer | 233 | 21.49 | 720 | 66.42 | **6.73 (4.76 – 9.50)** |

Abbreviations: OR, odds ratio; CI, confidence interval; *EGFR*, epidermal growth factor receptor;

^a^ Adjusted for gender, education, ethnicity, BMI, smoking status, family history of lung cancer, total energy intake, fruit, vegetable, and meat consumption.

^b^ Summed weekly consumption of fish, chicken or poultry, pork and other meat, and preserved meat.

^c^ Adjusted for gender, education, ethnicity, BMI, smoking status, family history of lung cancer, total energy intake, fruit, vegetable, and fish, chicken or poultry, pork and other meat, and preserved meat consumption.

^d^ As a large number of participants did not consume preserved meat, it was divided into non-consumer and consumer.

**Supplementary Table 6. Association between consumption of fruits, vegetables, and meat with risk of *EGFR*- lung cancer subtypes (age matched with caliper 0.2)**

| **Amount of food intake (Standard servings per week)** | **Controls**  **(N = 808)** | | ***EGFR*- Cases**  **(N = 808)** | | **Adjusted OR (95% CI)** |
| --- | --- | --- | --- | --- | --- |
|  | **n** | **%** | **n** | **%** |  |
| Fresh fruits ^a^ |  |  |  |  |  |
| Low (≤2.5) | 256 | 31.68 | 419 | 51.86 | 1 |
| Medium (>2.5 - ≤6.9) | 245 | 30.32 | 151 | 18.69 | **0.55 (0.36 – 0.82)** |
| High (>6.9) | 307 | 38.00 | 238 | 29.46 | 0.66 (0.44 – 1.00) |
| Vegetables ^a^ |  |  |  |  |  |
| Low (≤9) | 287 | 35.52 | 352 | 43.56 | 1 |
| Medium (>9 - ≤17) | 249 | 30.82 | 239 | 29.58 | 0.90 (0.61 – 1.33) |
| High (>17) | 272 | 33.66 | 217 | 26.86 | 0.88 (0.57 – 1.36) |
| Total Meat ^a,b^ |  |  |  |  |  |
| Low (≤5) | 304 | 37.62 | 180 | 22.28 | 1 |
| Medium (>5 - ≤9) | 255 | 31.56 | 300 | 37.13 | **2.06 (1.31 – 3.23)** |
| High (>9) | 249 | 30.82 | 328 | 40.59 | **3.35 (1.62 – 6.95)** |
| Fish ^c^ |  |  |  |  |  |
| Low (≤1.75) | 258 | 31.93 | 230 | 28.47 | 1 |
| Medium (>1.75 - ≤3.5) | 269 | 33.29 | 327 | 40.47 | 1.37 (0.83 – 2.25) |
| High (>3.5) | 281 | 34.78 | 251 | 31.06 | **2.73 (1.47 – 5.06)** |
| Chicken or Poultry ^c^ |  |  |  |  |  |
| Low (≤0.5) | 274 | 33.91 | 117 | 14.48 | 1 |
| Medium (>0.5 - ≤2.25) | 281 | 34.78 | 229 | 28.34 | 1.51 (0.87– 2.63) |
| High (>2.25) | 253 | 31.31 | 462 | 57.18 | 3.55 (1.98 –6.38) |
| Pork and other meat ^c^ |  |  |  |  |  |
| Low (≤0.46) | 265 | 32.80 | 144 | 17.82 | 1 |
| Medium (>0.46 - ≤2.25) | 360 | 44.55 | 212 | 26.24 | 0.62 (0.37 – 1.04) |
| High (>2.25) | 183 | 22.65 | 452 | 55.94 | **2.32 (1.12 – 4.84)** |
| Preserved meat ^c,d^ |  |  |  |  |  |
| Non-consumer | 640 | 79.21 | 257 | 31.81 | 1 |
| Consumer | 168 | 20.79 | 551 | 68.19 | **7.77 (4.84 – 12.49)** |

Abbreviations: OR, odds ratio; CI, confidence interval; *EGFR*, epidermal growth factor receptor;

^a^ Adjusted for gender, education, ethnicity, BMI, smoking status, family history of lung cancer, total energy intake, fruit, vegetable, and meat consumption.

^b^ Summed weekly consumption of fish, chicken or poultry, pork and other meat, and preserved meat.

^c^ Adjusted for gender, education, ethnicity, BMI, smoking status, family history of lung cancer, total energy intake, fruit, vegetable, and fish, chicken or poultry, pork and other meat, and preserved meat consumption.

^d^ As a large number of participants did not consume preserved meat, it was divided into non-consumer and consumer.
